# Functional Photoacoustic and Ultrasonic Assessment of Osteoporosis: A Clinical Feasibility Study

**DOI:** 10.34133/2020/1081540

**Published:** 2020-10-30

**Authors:** Ting Feng, Yunhao Zhu, Richard Morris, Kenneth M. Kozloff, Xueding Wang

**Affiliations:** ^1^ Department of Biomedical Engineering, University of Michigan Medical School, MI 48109, USA; ^2^ IF, LLC, WI 53589USA; ^3^ Department of Orthopaedic Surgery, University of Michigan Medical School, MI 48109, USA; ^4^ Department of Radiology, University of Michigan Medical School, MI 48109, USA

## Abstract

*Objective and Impact Statement*. To study the feasibility of combined functional photoacoustic (PA) and quantitative ultrasound (US) for diagnosis of osteoporosis *in vivo* based on the detection of chemical and microarchitecture (BMA) information in calcaneus bone. *Introduction*. Clinically available X-ray or US technologies for the diagnosis of osteoporosis do not report important parameters such as chemical information and BMA. With unique advantages, including good sensitivity to molecular and metabolic properties, PA bone assessment techniques hold a great potential for clinical translation. *Methods*. By performing multiwavelength PA measurements, the chemical information in the human calcaneus bone, including mineral, lipid, oxygenated-hemoglobin, and deoxygenated-hemoglobin, were assessed. In parallel, by performing PA spectrum analysis, the BMA as an important bone physical property was quantified. An unpaired t-test and a two-way ANOVA test were conducted to compare the outcomes from the two subject groups. *Results*. Multiwavelength PA measurement is capable of assessing the relative contents of several chemical components in the trabecular bone *in vivo*, including both minerals and organic materials such as oxygenated-hemoglobin, deoxygenated-hemoglobin, and lipid, which are relevant to metabolic activities and bone health. In addition, PA measurements of BMA show good correlations ($R^{2}$ up to 0.65) with DEXA. Both the chemical and microarchitectural measurements from PA techniques can differentiate the two subject groups. *Conclusion*. The results from this initial clinical study suggest that PA techniques, by providing additional chemical and microarchitecture information relevant to bone health, may lead to accurate and early diagnosis, as well as sensitive monitoring of the treatment of osteoporosis.

## 1. Introduction

Osteoporosis is a highly prevalent disease, affecting approximately 40% of women and 20% of men over the age of 50 years [1, 2]. Fragility fractures associated with osteoporosis result in large individual and societal costs. Hence, early and accurate detection of changes in bone quality and quantity is crucial for reducing the clinical and economic burdens associated with osteoporotic fractures.

Clinically available diagnostic technologies for osteoporosis rely on either X-ray or ultrasound (US) to assess bone mass. Dual energy X-ray absorptiometry (DXA or DEXA) is considered the clinical gold standard for assessing bone mineral density (BMD). DEXA, however, does not describe fracture risk completely, as it fails to report other important parameters such as bone microarchitecture (BMA), bone elastic properties, and chemical information. [3] Quantitative ultrasound (QUS) techniques provide a practical and low-cost surrogate for DEXA and have led to commercialized instruments for clinic [4, 5]. QUS assessment of bone structure and strength is mainly performed in the transmission mode and is based on the measurement of two key parameters including speed of sound (SOS) and broadband ultrasonic attenuation (BUA). These parameters are strongly correlated with BMD but are less reflective of BMA. [6, 7] As a result, after many years of development, QUS has not demonstrated superiority over DEXA and, despite its low-cost and safe implementation, still has limited use in clinic. [8]

The overall mechanical integrity of the bone arises from the quantity (mass) and structure (organization) of the bone, as well as the quality (material properties) of the bone tissue itself. The bone tissue is comprised of nonorganic mineral matrix as well as organic, proteinaceous component that is dominantly type I collagen. The mineral component of the bone contributes to the bone's stiffness, while the amount and organization of the organic phase is typically responsible for its toughness or postyield behavior. Loss of structure (i.e., loss of both mineral and organic materials) contributes to osteoporosis and subsequent fragility. Recent developments in magnetic resonance imaging (MRI) have demonstrated the feasibility and diagnostic relevance of characterizing not only bone microarchitecture but also bone metabolic processes at the molecular level. [9] The bone, as an organ, can be considered to also include bone marrow components–organic matrices that include lipids, proteins, vascularity, and blood cells. MRI has the potential to investigate several of these aspects of bone physiology that are not captured by DEXA, including marrow fat content, perfusion, and molecular diffusion. [10, 11] As BMD decreases, bone marrow fat content tends to increase while bone marrow perfusion indices decrease. [12–14] The correlation between marrow fat fraction obtained with magnetic resonance spectroscopy (MRS) and BMD obtained with DEXA has been reported as high as r=0.91. [15] Another study in the population-based Age Gene/Environment Susceptibility (AGES) cohort found that higher marrow fat assessed by MRS was correlated with lower trabecular BMD and prevalent vertebral fracture [16]. Bone perfusion is another physiological change reflecting bone health. In two independent studies on 90 male and 110 female subjects, respectively, perfusion indices from MRI were significantly decreased in osteoporotic subjects compared with osteopenic subjects or those with normal bone density [10]. Thus, with the capability to evaluate chemical properties in bone, MRI can provide additional diagnostic information beyond DEXA alone. However, the high cost and limited access of MRI make it infeasible to be used as a tool for screening and treatment monitoring of osteoporosis.

The laser-induced photoacoustic (PA) imaging and sensing technologies have drawn considerable attention in recent years. Based on the detection of light-induced ultrasonic signals which are much less scattered in biological tissues compared to light, PA imaging and sensing can present more spatial information in deep tissues than pure optical techniques. Hence, PA imaging, as a complement to established US imaging, has shown promise in many preclinical and clinical applications [17, 18]. The majority of previous adaptations of PA techniques were on soft tissues, and only a few attempts were made on the bones, likely due to the challenges in light delivery and ultrasonic detection in this mineralized hard tissue. Lashkari et al. used a dual backscattered US and PA radar system for the assessment of cortical and trabecular bone structures and density variations and demonstrated that PA and US could discriminate the changes associated with osteoporosis [19, 20]. Our group validated the feasibility of using PA spectral analysis to assess the microarchitecture of the trabecular bone through a study on a rat model of bone loss and preservation induced by ovariectomy and subsequent weekly dosing of zoledronic acid. [21] In another work utilizing the same animal models, we demonstrated that thermal PA measurement, based on the mechanism of temperature dependence of the Grüneisen parameter, can assess the BMD in the trabecular bone [22]. Steinberg et al. developed quantitative multispectral PA methods and examined them on fowl radius bones *in vitro*, indicating that PA measurements were correlated well with QUS counterparts, and the use of multispectral PA could provide additional functional information for detection of osteoporosis [23]. Cayla et al. introduce an optimized PA imaging technique to assess SO_2_ within the femoral bone marrow cavity through disease progression in a murine model. [24] All these previous studies, however, were conducted on *ex vivo* bone specimens or *in vivo* small animal models, while possible challenges for *in vivo* applications on human study, e.g., the light attenuation in overlying soft tissues, were not considered. More recently, Steinberg et al. have reported the first-in-human study using a low-cost and compact dual-wavelength PA system [25]. Although involving only a small cohort of healthy volunteers, this study showed the repeatability and accuracy of the PA method and its potential for early diagnosis of bone pathologies.

In the present study, for the first time to the best of our knowledge, a transmission mode multiwavelength PA and US combined system for measuring human calcaneus bones *in vivo* was developed. The feasibility of this system in detecting osteoporosis and characterizing bone health was tested via an initial clinical study involving 10 healthy volunteers and 10 patients with osteopenia or osteoporosis as confirmed by DEXA. We targeted the measurement of the calcaneus bone for several reasons. First, former research suggested that the calcaneus bone is the best external site for risk prediction of future spine and appendicular fractures due to its high percentage of the trabecular bone. [26, 27] Second, the human calcaneus bone has an easy access for noninvasive imaging and measurement by both light and ultrasound due to its limited covering by a thin layer of soft tissue. Third, the calcaneus bone is the target for many established QUS bone characterization techniques including those successfully commercialized and adopted by clinics, such as the GE Achilles Lunar ultrasonometer which also measures the calcaneus bone in the transmission mode. Hence, our developed transmission mode PA bone measurement technique can be integrated with an existing QUS system to achieve multimodality bone characterization. When working in the PA transmission mode, the light illumination and the ultrasound detection are at the opposite sides of the heel. With this arrangement, the large PA signals generated in the skin and soft tissues at the light illumination side of the heel arrive at the transducer later than the signals from the trabecular bone. Hence, these soft tissue signals and their reverberations can be easily separated from the targeted bone signals based on the difference in time of flight.

In this study, by measuring the heel using safe laser light at different wavelengths, we explored the feasibility of multiwavelength PA measurement in characterizing the chemical components in the trabecular part of the calcaneus bone. In addition, by quantitatively studying the power spectra of the PA signals from the trabecular part of the bone, we explored the feasibility of PA spectral analysis in characterizing bone microarchitecture. These quantitative PA measurements of the calcaneus bones from the two groups of subjects were compared, aimed at examining the hypothesis that the PA techniques can characterize both chemical information and microarchitecture relevant to bone health.

## 2. Results

### 2.1. DEXA Imaging and QUS Results

The DEXA imaging results from osteoporosis patients and controls are shown in Figures 1(a) and 1(b). Correlations between the stiffness indices from QUS and the BMD results from DEXA were studied, as shown in Figures 1(c)–1(f). An $R^{2}$ in the range of 0.45-0.65 were achieved between the QUS and the BMD results, depending on the location where the BMD were measured. This study based on commercial DEXA and QUS technologies confirmed the pathologic conditions of the osteoporotic patients as well as the difference between the two groups of human subjects.

Figure 1DEXA images and QUS results. (a) DEXA images showing spine and femur regions of interest. (b) DEXA imaging at the spine and the hip demonstrates significantly reduced BMD in the osteoporotic patients vs. controls. ${}^{**}$ stands for P<0.01, and ${}^{***}$ stands for P<0.001 in unpaired t-test comparing the results from the two subject groups (N=10 for osteoporosis group, N=10 for control group). (c) The bone stiffness indices acquired from the QUS device vs. the BMD results from the spine (c), total femur (d), femur neck (e), and femur upper neck (f), respectively, as quantified by DEXA.

**(a):**
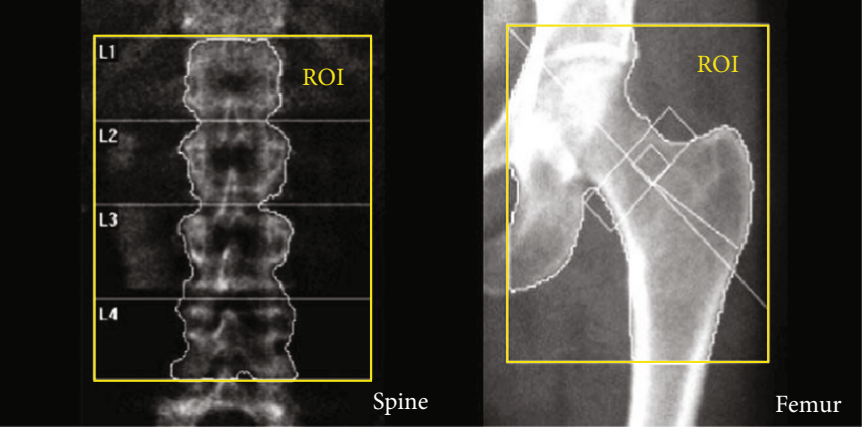

**(b):**
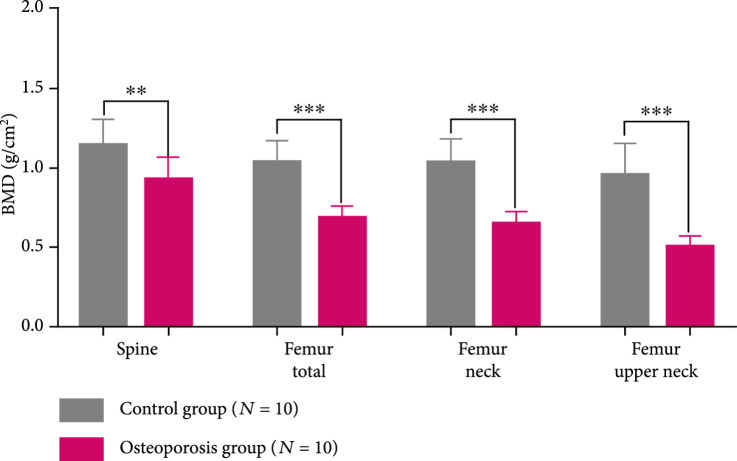

**(c):**
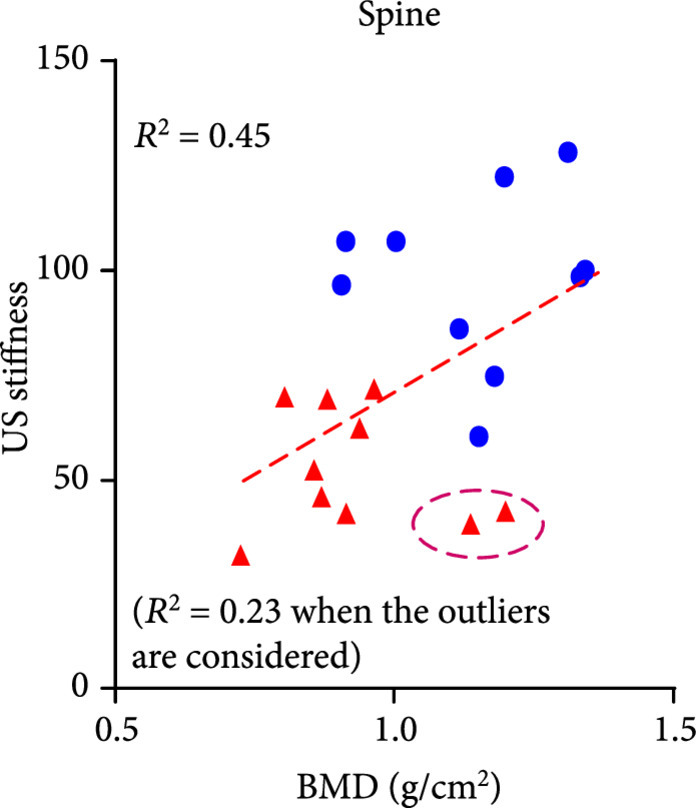

**(d):**
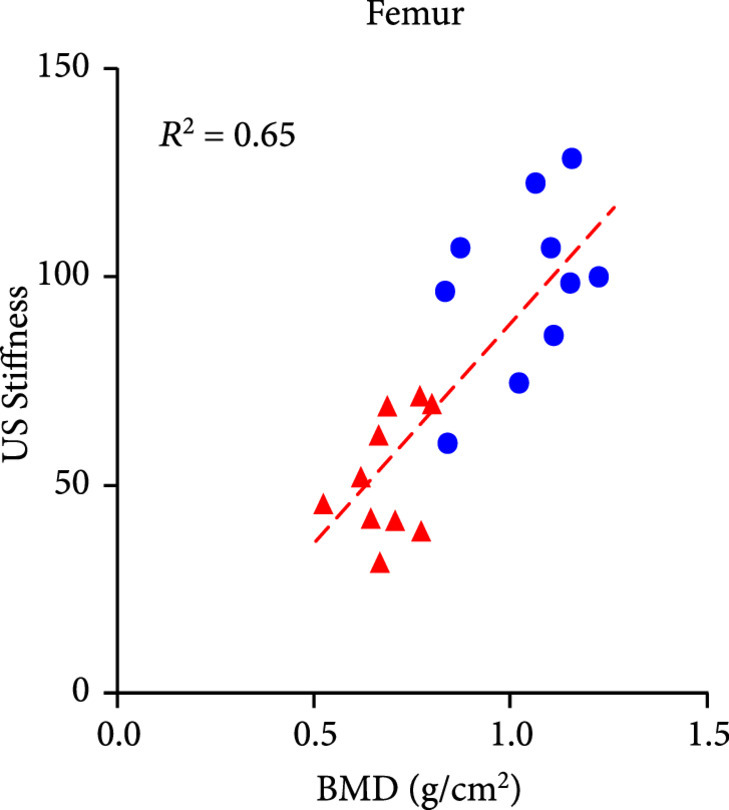

**(e):**
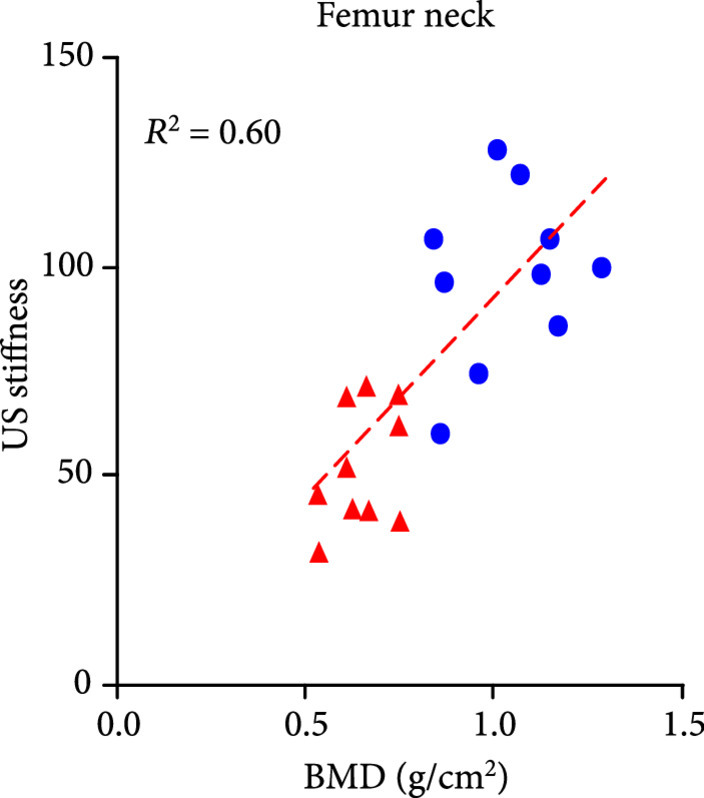

**(f):**
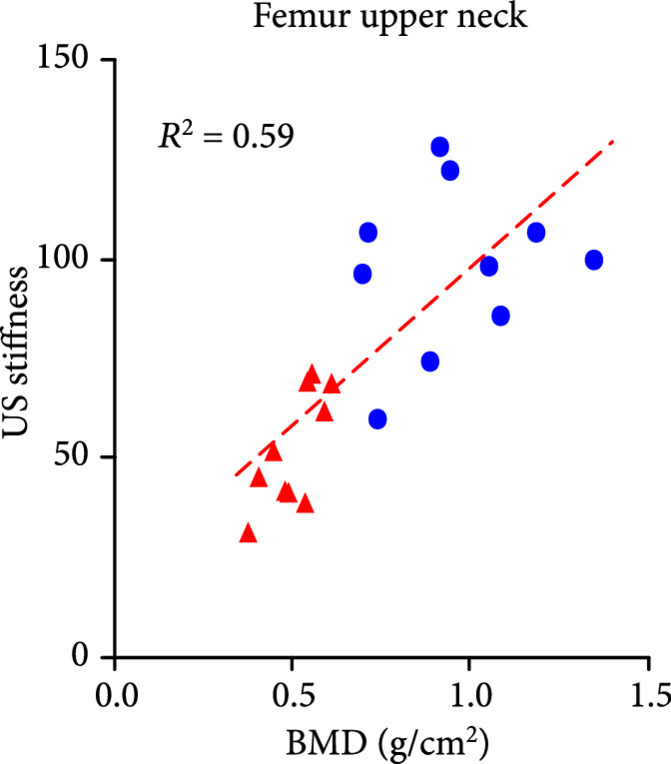

### 2.2. *In Vivo* Measurement of Bone Chemical Composition

The multiwavelength PA measurement results from the two groups of human subjects are shown in Figure 2. The relative optical absorption spectrum of each foot was obtained by using equation (10), where $\lambda_{0}$ was set at 800 nm. The two curves in Figure 2(a) are the average and the standard division of the relative optical absorption spectra of the calcaneus bones from the control group (N=8) and calcaneus bones from the osteoporosis group (N=10), respectively (without counting those with low average SNR in Table 1). By comparing to the curve from the control group, differences can be observed in the curve from the osteoporosis group, including (1) higher amplitude in the 700-750 nm range, demonstrating increased ratio of deoxy-hemoglobin in total blood content, and (2) higher amplitude at around 930 nm, demonstrating increased ratio of lipid content over blood content. These suggest that osteoporotic bones contain higher content of deoxy-hemoglobin (or lower oxygen saturation) and increased content of lipid, which match with the findings from a prior work [28]. To evaluate whether these two average optical absorption spectrum curves from the two subject groups have a statistically significant difference, a two-way ANOVA test was conducted by using the GraphPad Prism 7.0 software, which led to P<0.001.

Figure 2Multiwavelength PA measurement of the optical absorption spectrum of the trabecular bone in the human calcaneus *in vivo*. (a) The relative optical absorption spectra of the calcaneus bones calculated from multiwavelength PA measurements from the control group (N=8) and the osteoporosis group (N=10), respectively, each normalized at 800 nm wavelength. The solid curves show the average over the subjects in each group, and the shadow shows the standard deviation at each wavelength. (b) The optical absorption spectra of different chemical components in the trabecular bone, including deoxy-hemoglobin (Hb), oxy-hemoglobin (HbO_2_), lipid, and hydroxyapatite, which are the major contributors to the optical absorption of the trabecular bone in the optical spectrum of 690-950 nm. (c) The fitted optical absorption spectra from spectral unmixing by using the least-square regression method compared to the measured optical absorption spectra from an osteoporosis patient and a control volunteer, respectively. The quantified correlations $R^{2}$ were 0.97 and 0.99, respectively. (d) The spectral unmixing results showing the relative contents of hydroxyapatite, lipid, Hb, HbO_2_, and blood (i.e., Hb + HbO_2_), as well as the blood oxygen saturation (SaO_2_), of the two groups. ${}^{*}$ stands for P<0.05 in unpaired t-tests comparing the results from the two subject groups (N=10 for osteoporosis group, N=8 for control group).

**(a):**
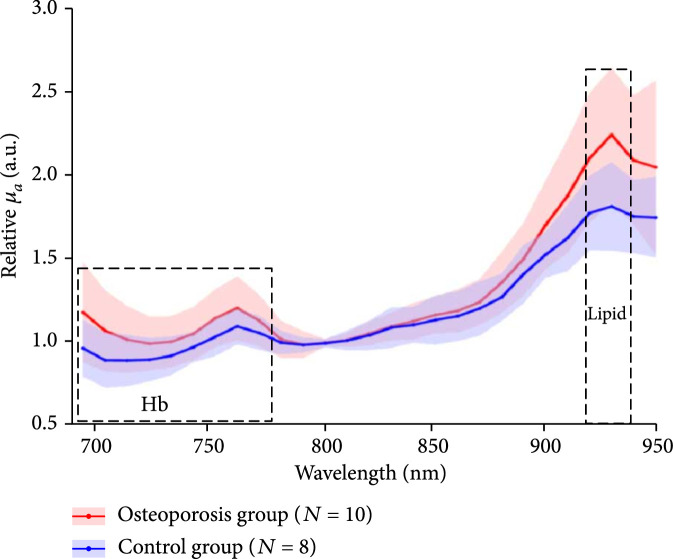

**(b):**
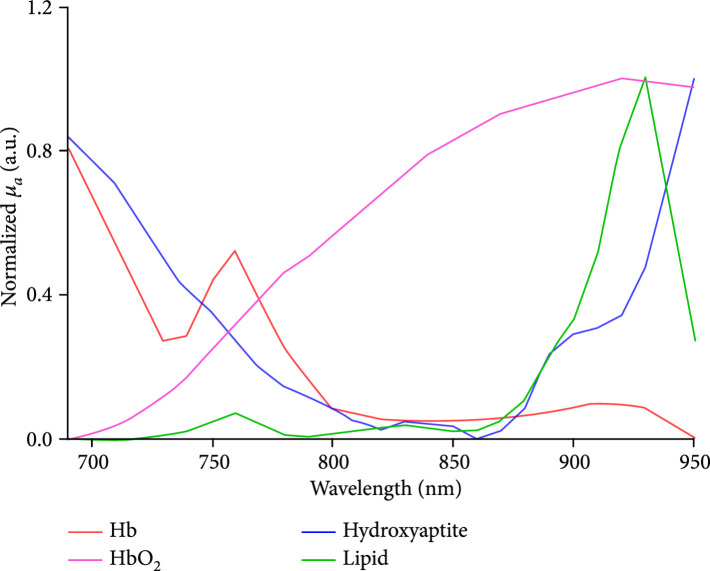

**(c):**
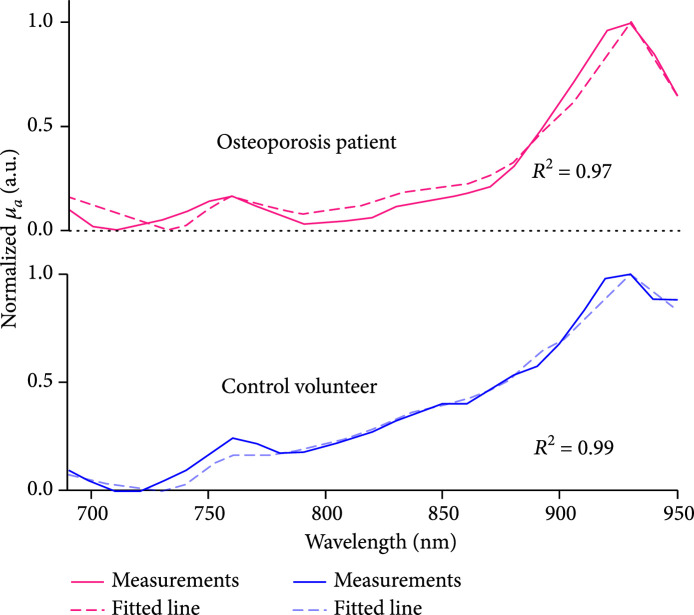

**(d):**
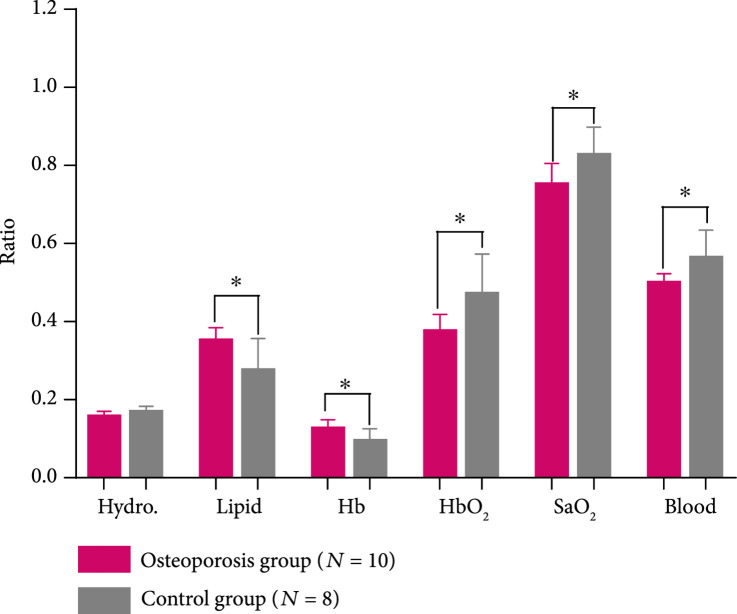

Table 1The averaged SNR of the PA signals from the calcaneus bone at all wavelengths for each foot of 10 patients (osteoporosis group) and 10 normal volunteers (control group).

**(a):** 

Osteoporosis group	Average SNR	
	Left	Right
P001	22.22	6.48
P002	18.28	8.38
P003	6.59	22.61
P004	*3.38*	15.45
P005	27.82	20.67
P006	9.43	22.68
P007	4.08	6.51
P008	6.53	*2.44*
P009	19.41	32.67
P010	7.50	16.12

**(b):** 

Control group	Average SNR	
	Left	Right
H001	18.23	5.33
H002	7.28	12.74
H003	24.05	7.15
H004	21.25	14.83
H005	*1.43*	*0.91*
H006	6.11	7.17
H007	5.32	4.41
H008	*1.06*	*1.77*
H009	13.68	12.96
H010	4.25	10.43

Due to the partial overlapping of the optical absorption spectra of hydroxyapatite and blood in the studied spectral range, detailed changes in chemical contents could not be observed directly from the curves in Figure 2(a). To derive the quantitative changes in the contents of all chemical components which are the major contributors to the optical absorption spectrum of the calcaneus bone, spectral unmixing based on the least-square regression method was conducted. [29] The four major chromophores considered in the spectral unmixing were deoxy-hemoglobin, oxy-hemoglobin, lipid, and hydroxyapatite, with their optical absorption spectra presented in Figure 2(b). [22, 30] When performing the spectral unmixing, the relative contents of hydroxyapatite, lipid, deoxy-hemoglobin, and oxy-hemoglobin were set in the range of 0-25%, 0-70%, 0-30%, and 0-50%, respectively, according to the findings in the literature [28]. Setting a range for relative content of each chromophore can speed up the spectral unmixing process and improve accuracy. Satisfactory accuracy in spectral unmixing was achieved, as demonstrated in Figure 2(c) where two fitted spectra by using the least-square regression method show a good match with the measured optical absorption spectra from an osteoporosis patient and a control volunteer, respectively. For both of these two cases, good correlation between the measured spectrum and the fitted spectrum was achieved ($R^{2}=0.97$ and 0.99, respectively). Of note, the spectral unmixing here did not lead to the measurements of molar concentrations of the chemical components, because the data in Figure 2(b) utilized in spectral unmixing were not the molar extinction coefficients. The relative content derived from the spectral unmixing reflects the weight of each optically absorbing chemical component in the PA-measured optical absorption spectrum of the bone.

By performing spectral unmixing, the relative contents of the key chemical components in each foot were obtained. Because each patient was counted as one data point in the statistical analyses for Figure 2, the chemical measurements from the left and right feet of each patient were averaged first. The means and the standard deviations of the quantified chemical contents in the calcaneus bones from the osteoporosis patients (N=10) and the control volunteers (N=8) (without counting those with low average SNR in Table 1) are compared in Figure 2(d). To evaluate whether each of the differences in chemical properties between the two subject groups has statistical significance, an unpaired two-tailed independent samples t-test (with Welch's correction in cases of unequal variances) was conducted by using the GraphPad Prism 7.0 software. Compared to the control group, the chemical changes in the osteoporosis group showing statistical significance include the increased lipid content, the decreased blood content, and decreased blood oxygen saturation. The latter two are the combined effect of the increased contents of deoxy-hemoglobin and the decreased content of oxy-hemoglobin in the osteoporosis bones. These noticed chemical changes in osteoporosis bones match with the findings reported in previous publications [9, 10, 11, 12, 13, 15]. Compared to the control group, the calcaneus bones in the osteoporosis group also show decreased hydroxyapatite content, which, however, is not statistically significant.

### 2.3. *In Vivo* Measurement of Bone Microarchitecture

With RF PA signal from each calcaneus bone acquired at each optical wavelength, the PSD was derived using equation (13). Figure 3(a) shows the average and the standard deviations of the PSD curves of the calcaneus bones from the control group (N=8) and the osteoporosis group (N=10), respectively, all acquired at 800 nm wavelength. Figures 3(b) and 3(c) are the PSD curves after compensating the ultrasound attenuation. As shown in Figures 3(b) and 3(c), the osteoporosis group has lower high-frequency components in comparison with the control group. This can be explained by the fact that the osteoporosis bone has larger porosity which is filled by marrow, and spatially distributed marrow with larger scales generates PA signals with lower frequency. To evaluate whether these two average PSD curves have statistically significant difference, a two-way ANOVA test was conducted by using the GraphPad Prism 7.0 software, leading to P<0.001. The linear fitting of the average PSD curves from 0.1 MHz to 1 MHz, as shown in Figure 3(b), led to the spectral parameter *slopes* of 0.11 dB/MHz and 0.20 dB/MHz for the two groups, respectively. In Figure 3(c), by computing the areas under each PSD curve from 0.1 MHz to 1 MHz, *weighted frequencies* of 72.78 dB·MHz and 138.62 dB·MHz were obtained for the two groups, respectively. Both *slope* and *weighted frequency*, as quantified PA spectral parameters, quantitatively present the weaker high-frequency components in the PA signals from the osteoporosis bones at 800 nm laser wavelength.

Figure 3The normalized power spectrum density (PSD) of the RF PA signals from the calcaneus bones in the osteoporosis group (N=10) vs. the control group (N=8). The shadows show the standard deviations. (a) The PSD curves of the PA signals from the osteoporosis group and the control group before compensating the ultrasound attenuation. (b, c) The PSD curves of the PA signals from the osteoporosis group and the control group after compensating the ultrasound attenuation. In (b), the linear fitting over the frequency range of 0.1-1 MHz led to quantified spectral parameter *slopes* of 0.11 dB/MHz and 0.20 dB/MHz for the osteoporosis group and the control group, respectively. In (c), the integrated areas under the two curves over the frequency range of 0.1-1 MHz led to quantified spectral parameter *weighted frequencies* of 72.78 dB·MHz and 138.62 dB·MHz for the osteoporosis group and the control group, respectively.

**(a):**
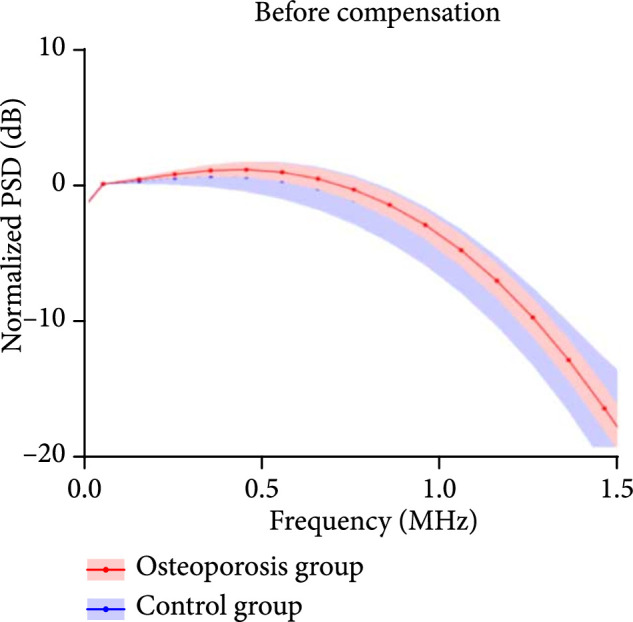

**(b):**
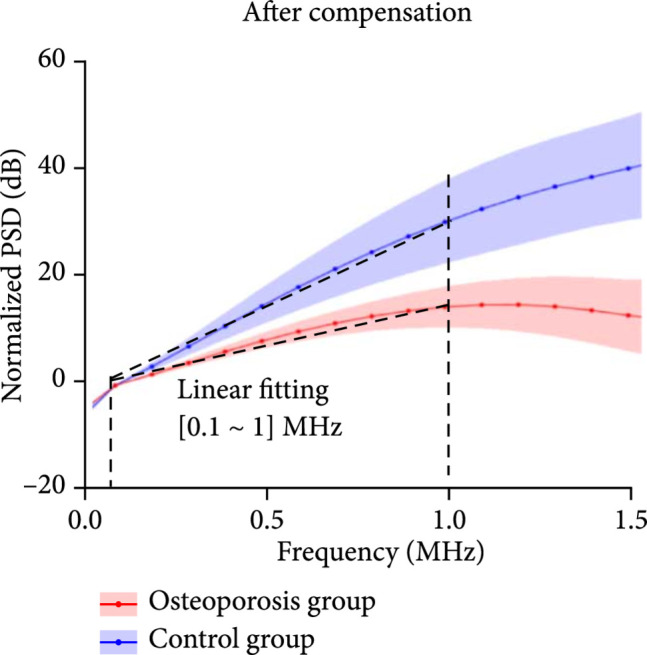

**(c):**
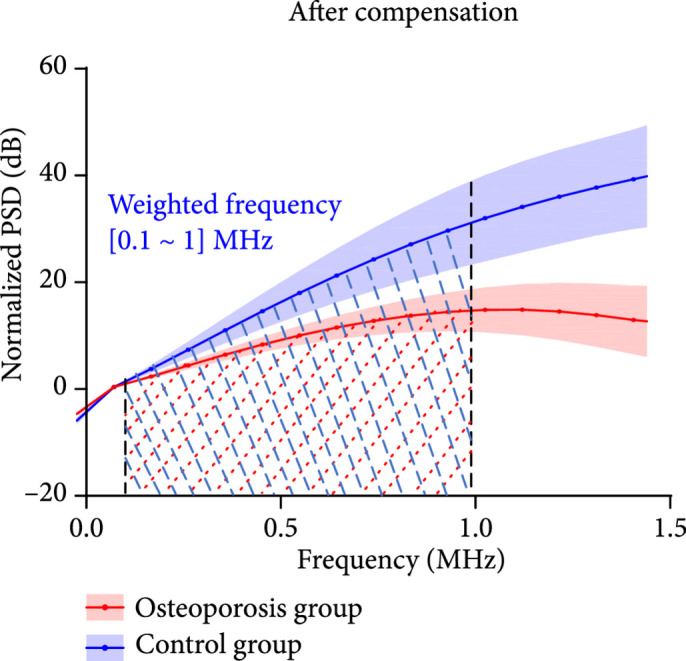

**Figure 4:**
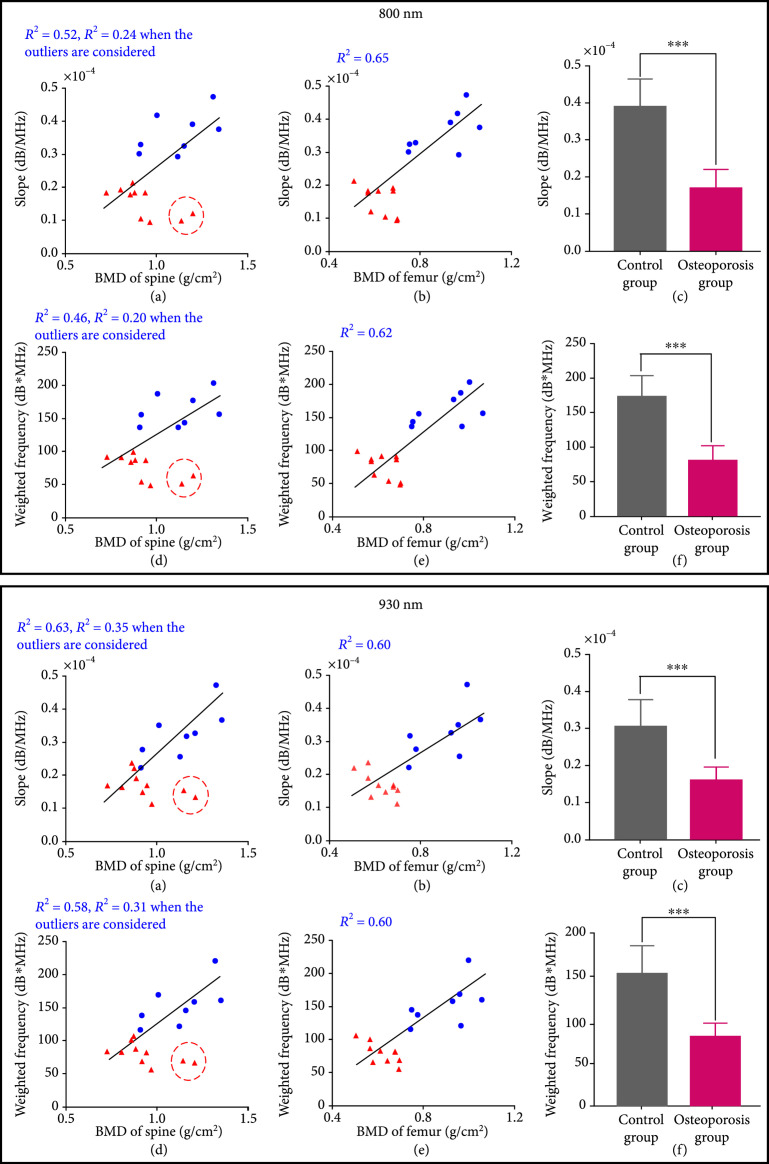
PA spectral analysis results of the control group (N=8) vs. the osteoporosis group (N=10). The upper and the lower panels are for the results acquired at the 800 nm and 930 nm laser wavelengths, respectively. In each panel, (a) shows the PA spectral parameter *slopes* as a function of the BMD of the spine measured by DEXA, (b) shows the PA spectral parameter *slopes* as a function of the BMD of the total femur measured by DEXA, (c) shows the averages and the standard deviations of the quantified *slopes* from the two groups, (d) shows the PA spectral parameter *weighted frequencies* as a function of the BMD of the spine measured by DEXA, (e) shows the PA spectral parameter *weighted frequencies* as a function of the BMD of the total femur measured by DEXA, and (f) shows the averages and the standard deviations of the quantified *weighted frequencies* from the two groups. The PA spectral parameters from the control group and the osteoporosis group are shown in blue circles and red rectangles, respectively. ${}^{***}$ stands for P<0.001 in the unpaired t-test comparing the spectral parameters from the two groups. The two outliers in (a) and (d) in each panel were resulting from aberrant DEXA analyses and are reasonable to be excluded from the analyses.

We studied the correlations between the PA spectral parameters and the BMD results from DEXA and also examined if there were any statistically significant difference in the PA spectral parameters between the two subject groups, as shown in Figure 4. Because each patient was counted as one data point in the statistical analyses for Figure 4, the quantified PA spectral parameters from the left and the right feet of each patient were averaged first. For the results acquired at each of the two laser wavelengths, each of the quantified PA spectral parameters, including the *slope* and the *weighted frequency*, was correlated with the BMD of spine and the BMD of femur from DEXA, as shown in (a), (b), (d), and (e) in each panel of Figure 4. Satisfactory correlations of $R^{2}$ up to 0.65 (between the *slope* at 800 nm and the BMD of femur) were observed, with a range of 0.46-0.65, depending on the spectral parameter and the location for DEXA measurements. We can see two outliers in (a) and (d) in each panel of Figure 4. These two subjects demonstrated significant underprediction of PA spectral parameters *slope* and *weighted frequency* by spinal BMD compared to hip BMD from the same subjects. Post hoc assessment of DEXA images revealed spinal abnormalities including variance in vertebral height loss, mild scoliosis, and sclerosis that precluded an accurate BMD assessment at the spine. Therefore, in this case, PA spectral parameters were able to reveal systemic osteoporosis at the heel that was verified in the hip but subject to error of interpretation in the spine.

For the PA results acquired at each laser wavelength, the average and the standard deviation of each spectral parameter were calculated for each group of subjects, as shown in (c) and (f) in each panel of Figure 4. To evaluate whether each of the spectral parameters can differentiate the two groups with statistical significance, an unpaired two-tailed independent samples t-test (with Welch's correction in cases of unequal variances) was conducted by using the GraphPad Prism 7.0 software. Each statistical analysis led to P<0.001, demonstrating that each of the PA spectral parameter (*slope* and *weighted frequency*) acquired at each of the two laser wavelengths (800 nm and 930 nm), by assessing the changes in bone microarchitecture, can differentiate the control group and the osteoporosis group.

## 3. Discussion

For the first time, to the best of our knowledge, a transmission mode multiwavelength PA and US combined system for measuring human calcaneus bones *in vivo* was developed, and its performance in diagnosis of osteoporosis was validated via an initial clinical study involving 10 healthy volunteers and 10 patients with osteopenia or osteoporosis. This label-free and noninvasive PA and US dual-modality system for measuring human calcaneus bone *in vivo* was built on a commercial GE Achilles Lunar ultrasonometer without affecting its original QUS functions. The ultrasonic parameters of the calcaneus bone, including BUA and SOS, acquired during the US mode were used to compensate for the ultrasound attenuation of the PA signals in the bone, which was an essential step for quantitatively assessing the bone in the PA mode. After the compensation, multiwavelength PA signals from the bone acquired over a spectral range of 690-950 nm were used to assess the contents of the optically absorbing chemical components in the trabecular bone, including oxy-hemoglobin, deoxy-hemoglobin, lipid, and hydroxyapatite. In addition, quantitative analyses of the PA signals in the frequency domain were performed to evaluate the microarchitecture information in the bone. The quantified PA spectral parameters, including *slope* and *weighted frequency* which can reflect the bone microarchitecture, were correlated with the BMD measured by DEXA as a clinical gold standard. The quantified PA measurements, including both chemical changes and microarchitecture changes, were compared between the two subject groups to determine the feasibility of each measurement in differentiating the osteoporotic subjects from the controls.

The results from this initial clinical study support several conclusions: (1) when applying laser fluence within the ANSI safety limit, PA measurement of a human heel in the transmission mode can detect the signal from the trabecular part of the calcaneus bone with satisfactory SNR; (2) when combined with the spectral unmixing method, multiwavelength PA measurement of a calcaneus bone over the optical spectrum of 690-950 nm is capable of assessing the relative contents of several chemical components in the trabecular bone, including both minerals (hydroxyapatite) and organic materials such as oxy-hemoglobin, deoxy-hemoglobin, and lipid, which are relevant to metabolic activities and bone health; (3) the spectral analysis of the RF PA signals acquired at different laser wavelengths leads to quantified spectral parameters, including *slope* and *weighted frequency*, which can evaluate bone microarchitectures composed by different chemical materials; and (4) the quantified parameters from the noninvasive PA measurements of human calcaneus bone, including both the bone chemical contents from the multispectral PA measurement and the bone microarchitectures from the PA spectral analysis, are capable of differentiating the osteoporotic patients from the control subjects.

The presented clinical study also has some limitations. First, the PA and US dual-modality system designed in this study was for transmission mode measurement of the calcaneus bone only and could not measure the human spine, femur, or hip directly–sites of high fracture rates in osteoporosis. However, as osteoporosis is a systemic condition, the pathologic condition measured from the calcaneus bone is highly correlated with the BMD assessed by the gold-standard DEXA at the spine, femur, and hip. [31] Second, working with the current system and laser fluence within the ANSI safety limits, several subjects with large calcaneus bones and thick overlying soft tissues turned out to be difficult in PA measurement, as reflected by the relatively low SNR in these cases. The measurement of difficult cases can be improved in the future by optimizing the light illumination pattern on the skin and the sensitivity of the ultrasound detection system. Third, in this initial clinical study, we only tested a small number of subjects (i.e., 10 normal volunteers and 10 osteoporosis patients) at the two extreme situations. In the future, additional clinical data should be collected and analyzed on a large group of subjects with a broad age range so that we can assess population-based differences in PA parameters across a wide range of ages. Fourth, although the initial pressure generated in the bone after the light absorption is broadband, due to the strong acoustic attenuation in the bone especially at high frequency, most of the PA signals received by the transducer are lower than 1 MHz. Therefore, to enhance the sensitivity in PA signal detection, the transducer used in this study had a relatively low center frequency of 0.5 MHz. For the same reason, only the spectrum range of 0.1-1 MHz was considered in PA spectral analysis. However, we also realize that higher frequency PA signals can carry more bone microarchitecture information, as reported in our previous paper [21]. Hence, one of the focuses in future work would be studying the sensitivity limitation of the detecting transducer as functions of the depth in the bone and the center frequency of the transducer.

Despite these limitations, this study successfully proved the feasibility of using the emerging PA techniques to assess the chemical and microarchitecture information in human calcaneus bone *in vivo*, noninvasively. In comparison with established imaging modalities such as DEXA, CT, and MRI, the presented PA-US dual-modality bone assessment method has many advantages such as target specific, quantitative, nonionizing, noninvasive, low cost, and patient and operator friendly. In addition, current skeletal imaging techniques fail to access the spatial information of organ-level cellular activity and chemical composition. Up until now, measuring the chemical changes in the bone matrix that have been associated with bone disease is limited primarily to *ex vivo* destructive testing of explanted bone samples. The development of PA techniques as a way to accurately quantify not only bone mass or microstructure but also organ-level chemical and molecular changes, such as lipid content, blood content (perfusion), and blood oxygenation, may allow for early identification of the changes in bone metabolism and quality. With all these unique advantages, the new PA techniques hold potential for quick translation to clinic. A PA and US dual-modality device may offer a combination of physical, chemical, and molecular biomarkers reflecting different aspects of bone health and may work together for describing early disease progress, evaluating early treatment response and predicting treatment outcome.

## 4. Materials and Methods

### 4.1. Human Subjects and Study Approval

For this initial clinical study, two groups of subjects were recruited. In the control group, 10 young, healthy female volunteers aged 20 to 29 years old were enrolled. The patient group including 10 female subjects aged 50 years or older was recruited through the University of Michigan Department of Orthopaedic Surgery Fragility Fracture Clinic. All subjects in the patient group were diagnosed with osteopenia or osteoporosis, as confirmed by DEXA. Subjects in the control group also received DEXA. All procedures in this study were approved by the Institutional Review Board of the University of Michigan School of Medicine (IRB: HUM00105987, assessment of bone health with light and sound. PI: Kozloff). The written informed consent was received from each participant prior to inclusion in the study.

### 4.2. PA and US Dual-Modality System

**Figure 5:**
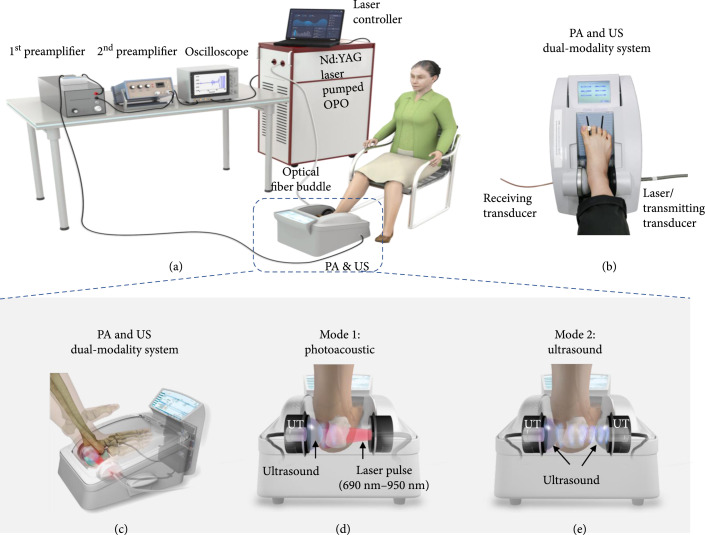
(a) Setup of the whole PA and US dual-modality bone characterization system. (b) Photograph showing the measurement on a human heel. (c–e) The concept of a PA and US dual-modality bone characterization system which can switch between the established QUS mode and the new PA mode.

The PA and US dual-modality bone measurement system shown in Figure 5 was built on a GE Achilles Lunar QUS device. All the transducers used in this system, including the receiving and the transmitting transducers in the QUS mode and the receiving transducer in the PA mode, are the same model and are unfocused, 1-inch diameter, and working at a low center frequency of 0.5 MHz with a -20 dB bandwidth of 0.19-0.74 MHz. These transducers were custom manufactured for the GE Achilles Lunar QUS devices. When working in the original US mode, this system is capable of providing QUS parameters of the calcaneus bone, including SOS, BUA, stiffness index, and T-score. In this study, the US stiffness index of each human calcaneus bone was acquired, and the results from the osteoporotic patients and the controls were compared. In addition, the spine, total femur, femoral neck, and femoral upper neck of each subject were imaged using a commercial DEXA machine (GE Lunar iDXA bone densitometer).

When working in the PA mode, the transmitting transducer was replaced by a bundle of optical fibers for delivery of laser light. The input end of the fiber bundle was connected to a Nd : YAG laser pumped OPO (Phocus MOBILE, OPOTEK Inc.). This laser system works at a repetition rate of 10 Hz, with a pulse width of 5 ns, and a continuous tuning range from 690 nm to 950 nm. The energy output from the laser can be precisely controlled. In addition, by controlling the distance between the output end of the fiber bundle and the surface of the skin, we can also control the total area of the light illumination on the skin surface. To ensure that the laser fluence illuminated on the skin surface was less than the ANSI safety limit, we measured the total energy delivered at the output end of the fiber bundle and the illumination area on the skin surface before each measurement on human subjects. For example, at 750 nm wavelength, a total energy of 60 mJ illuminated on a circular area with a diameter of 2 cm led to light fluence of 19.1 mJ/cm^2^ which was below the ANSI safety limit at this wavelength [32]. To evaluate different chemical components in the trabecular bone, including oxy-hemoglobin, deoxy-hemoglobin, lipid, and hydroxyapatite, which are the major contributors to the optical absorption of the trabecular bone in the studied spectral range, multiwavelength PA measurements were acquired from 690 nm to 950 nm with a constant step size of 10 nm. Within this optical spectrum, deoxy-hemoglobin has an absorption peak around 760 nm, oxy-hemoglobin has strong absorption at 900 nm, and lipid has an absorption peak at 930 nm. The hydroxyapatite has no obvious absorption peak in the spectrum from 690 nm to 950 nm and has a bottom around 850 nm. Laser-induced PA signals from the calcaneus bone were detected by the receiving transducer and were amplified first by 46 dB using a low-noise preamplifier (NF, SA-220F5) and then by an additional 40 dB using another preamplifier (5072PR, Panametrics). The PA signals, after a total of 86 dB amplification, were averaged over 150 laser pulses before being collected by an oscilloscope (Tektronix MSO54). All these steps helped to enhance the signal-noise-ratio (SNR) which was crucial for detecting the weak PA signals from the human calcaneus bone *in vivo*.

### 4.3. Detection and Compensation of PA Signal from Calcaneus Bone

**Figure 6:**
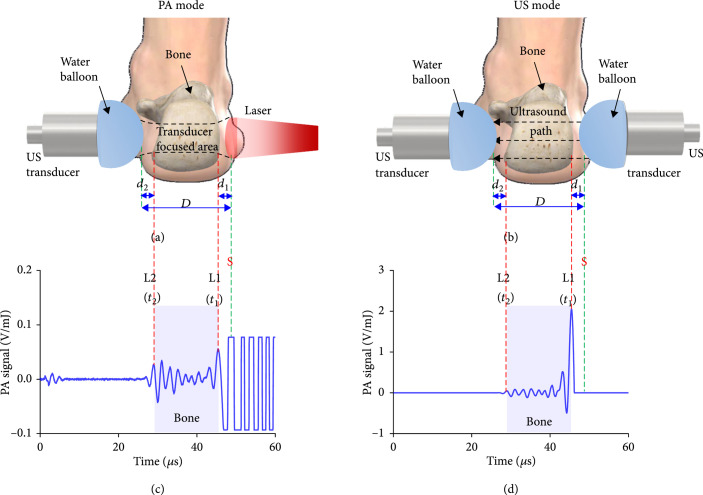
Detection of the PA signal from the human calcaneus bone *in vivo*. (a) The schematic diagram of the PA measurement of a human heel. (b) The schematic diagram of the QUS measurement of a human heel. (c, d) The calcaneus bone PA signal before and after the compensation of the ultrasound attenuation, respectively. The signal marked between the two red dashed lines is from the calcaneus bone, where the right and the left boundaries are marked by L1 and L2, respectively. The large and saturated signal at the right side of the green dashed line marked with "S" is from the soft tissue covering the bone.

Both feet of each subject were measured using the US and PA dual-modality system showing in Figure 6. Both the PA and US measurements were in the transmission mode, as shown in Figures 6(a) and 6(b), respectively. For transmission mode PA measurement, the light illumination and the ultrasound detection were at the opposite sides of the heel with the transducer and the laser beam coaxially aligned. This enabled the use of QUS parameters along the same pathway in the bone to calibrate PA signals. An example of the PA signal received from the human calcaneus bone *in vivo* is shown in Figure 6(c), where the large and saturated PA signal marked by "S" was generated by the soft tissue covering the bone, while the signal near the transducer marked between the two red dashed lines came from the bone. The right and left boundary of the PA signal generated by the bone was marked by L1 and L2, respectively.

For each of the two subject groups (osteoporosis group and control group), the 20 feet of the 10 subjects were measured. For each foot, the SNR of the PA signal from the calcaneus bone was calculated at each optical wavelength, and then, the results for all the wavelengths in the spectral region of 690-950 nm were averaged, as shown in Table 1. For some subjects, the average SNR for one foot or both feet was lower than 4 (as the data marked in italic). The PA measurements from these feet were not considered in any of the later analyses.

Before being received by the transducer, the PA signal experienced strong attenuation in the bone. The PA signal generated by the bone tissue between L1 and L2 and received by the US transducer can be expressed as

(1)pt =k∙Γ∙μa∙e−αs∙d2soft tissue∙e−αb∙t−t2∙cbbone∙e−μeffs∙d1soft tissue∙e−μeffb∙t1−t∙cbbone∙I0,

where Γ is the Grüneisen parameter which can be expressed as $\Gamma =\beta ∙c^{2}/C_{p}$, where β is the thermal coefficient of volume expansion, c is the SOS of tissue, and $C_{p}$ is the heat capacity at constant pressure; $\mu_{a}$ is optical absorption of bone tissue; $I_{0}$ is the light fluence generated by the laser; k is a constant accounting for the impulse response of the detecting system; $\alpha_{s}$ and $\alpha_{b}$ are the ultrasound attenuation coefficients in the soft tissue and the bone, respectively; $\mu_{\text{effs}}$ and $\mu_{\text{effb}}$ are the effective optical attenuations in the soft tissue and the bone, respectively; $t_{1}$ and $t_{2}$ are the signal arriving time of the location L1 and L2, respectively; $d_{1}$ and $d_{2}$ are thickness of the soft tissue at the left and right sides of the bone, respectively; $c_{b}$ is the speed of sound in the bone; ${\left(e^{-\alpha_{s}∙d_{2}}\right)}_{\text{soft}\;\text{tissue}}$ and ${\left(e^{-\alpha_{b}∙\left(t-t_{2}\right)∙c_{b}}\right)}_{\text{bone}}$ are the ultrasound attenuation of the PA signal in the soft tissue and the bone, respectively; $\;{\left(e^{-\mu_{\text{eff}}∙d_{1}}\right)}_{\text{soft}\;\text{tissue}}$ and ${\left(e^{-\mu_{\text{effb}}∙\left(t_{1}-t\right)∙c_{b}}\right)}_{\text{bone}}$ are the light attenuation in the soft tissue and the bone, respectively.

Because the ultrasound attenuation in the soft tissue is very low (about 0.5-1.0 dB/MHz·cm) for the 0.5 MHz PA signal [33, 34], it can be ignored. The ultrasound attenuation in the bone can be estimated via the US and PA dual-modality setting of the system. The GE Achilles Lunar allowed the estimation of the ultrasound parameters including BUA and SOS in the calcaneus bone. Of note, because the cortical part of human calcaneus bone is very thin while the cancellous and the marrow in the trabecular part of the bone cannot be spatially resolved at the working ultrasound frequency centered at 0.5 MHz, the SOS estimated by QUS is an average of the cortical, cancellous, and marrow parts of the calcaneus bone. In addition, we also estimated the total heel thickness D using a caliper. With these parameters, the PA signal at each location in the bone was compensated for ultrasound attenuation using the following equation:

(2)pct=pt∙10BUA∙Fc∙SOS∙t−t2/20∙D−d1+d2,

where $p_{c}\left(t\right)$ was the amplitude of the PA signal after compensation, $F_{c}\;$was the center frequency of the transducer which was 0.5 MHz, and $d_{1}$ and $d_{2}$ can be approximated by using empirical formula of 0.3×BMI+1.4. [35, 36] The PA signals before and after the compensation of the ultrasound attenuation are compared in Figures 6(c) and 6(d). In the signal after compensation, the amplitude at L1, which is from the bone at the light illumination side, is much larger than the amplitude at L2, which is from the bone at the ultrasound detection side. This is reasonable, and it is caused by the strong optical attenuation in the bone.

### 4.4. Multiwavelength PA Measurement of Bone Chemical Composition

By using the laser wavelengths from 690 nm to 950 nm with a constant step size of 10 nm, we conducted the multiwavelength PA measurements of the calcaneus bones of each subject. The PA signal of the bone after ignoring the ultrasound attenuation in the soft tissue and the compensation of ultrasound attention in the bone can be expressed as:

(3)pct =k∙Γ∙μa∙e−μeffs∙d1soft tissue∙e−μeffb∙t1−t∙cbbone∙I0.

We assume that both the optical absorption and the Grüneisen parameter in the bone are homogenous, i.e., $\Gamma \left(\text{L}1\right)=\Gamma \left(\text{L}2\right)\;$and $\mu_{a}\left(\text{L}1\right)=\mu_{a}\left(L2\right)$. The parameters including k, Γ, $\mu_{a}$, and ${\left(e^{-\mu_{\text{effs}}∙d_{1}}\right)}_{\text{soft}\;\text{tissue}}$ in equation (3) can be removed by dividing the PA amplitudes $p\left(t_{2}\right)$ and $p\left(t_{1}\right)$ at the locations L2 and L1:

(4)∆p=pct2pct1=k∙ΓL2∙μaL2∙e−μeffs∙d1soft tissue∙e−μeffb∙t1−t2∙cbbonek∙ΓL1∙μaL1∙e−μeffs∙d1soft tissue∙e−μeffb∙t1−t1∙cbbone=e−μeffb∙t1−t2∙cbbone.

Equation (4) is a function of the laser wavelength and can be expressed as

(5)∆pλ=e−μeffbλ∙t1−t2∙cb.

From this equation, we can get the $\mu_{\text{effb}}\left(\lambda \right)$ by using

(6)μeffbλ=ln∆pλt2−t1∙cb.

In this equation, $\left(t_{2}-t_{1}\right)∙c_{b}$ is independent on the wavelength and can be removed by dividing $\mu_{\text{effb}}\left(\lambda \right)$ to the one at the reference wavelength of $\lambda_{0}$:

(7)μeffbλμeffbλ0=ln∆pλ/t2−t1∙cbln∆pλ0/t2−t1∙cb=ln∆pλln∆pλ0=lnpct2/pct1λlnpct2/pct1λ0.

$\mu_{\text{effb}}\left(\lambda \right)$ is a function of the optical absorption coefficient $\mu_{a}\left(\lambda \right)$ and the optical scattering coefficient $\mu_{s}^{\prime }\left(\lambda \right)$, i.e., $\mu_{\text{effb}}\left(\lambda \right)=\sqrt{3∙\mu_{a}\left(\lambda \right)∙\left(\mu_{a}\left(\lambda \right)+\mu_{s}^{\prime }\left(\lambda \right)\right)}$. In the spectral range of 690-950 nm, $\mu_{a}\left(\lambda \right)$ is much lower than $\mu_{s}^{\prime }\left(\lambda \right)$, and hence, equation (7) can be simplified as

(8)μeffbλμeffbλ0=μaλ∙μs′λμaλ0∙μs′λ0.

Compared to $\mu_{a}\left(\lambda \right)$ which has large fluctuations in the spectrum of 690-950 nm, $\mu_{s}^{\prime }\left(\lambda \right)$ in the bone is flat in this optical spectrum, as reported in the literature [28]. Approximated to the empirical power law derived from the Mie theory, $\mu_{s}^{\prime }\left(\lambda \right)=\text{a}∙{\left(\lambda /500\right)}^{-b}$, and hence, $\sqrt{\mu_{s}^{\prime }\left(\lambda \right)}/\sqrt{\mu_{s}^{\prime }\left(\lambda_{0}\right)}$ can be simplified as$\;\sqrt{{\left(\lambda /\lambda_{0}\right)}^{-b}}$, where b in the calcaneus bone is around 0.67-0.73 [28, 30]. In the studied spectral range, the change in b leads to a small variation of $\sqrt{{\left(\lambda /\lambda_{0}\right)}^{-b}}$ less than 1%. Therefore, it is reasonable to assume b to be 0.7 (i.e., the median in the range of 0.67-0.73), and $\sqrt{\mu_{s}^{\prime }\left(\lambda \right)}/\sqrt{\mu_{s}^{\prime }\left(\lambda_{0}\right)}$ can be expressed as $\sqrt{{\left(\lambda /\lambda_{0}\right)}^{-0.7}}$. Then, equation (8) can be simplified as

(9)μeffbλμeffbλ0≈μaλμaλ0∙λλ0−0.7.

Conducting square at both sides of equation (9), we have

(10)μaλμaλ0≈μeffbλμeffbλ02∙λλ00.7=lnpct2/pct1λlnpct2/pct1λ02∙λλ00.7.

Equation (10) indicates that by performing multiwavelength PA measurement of a human heel, we can measure the relative optical absorption spectrum in the trabecular bone. In our experiment, $p\left(t_{1}\right)$ at each wavelength was considered the peak PA amplitude at the location of L1. Since the PA signal at L2 was weak, to improve the accuracy in multiwavelength PA measurement, $p\left(t_{2}\right)$ was considered the average of the absolute PA signal amplitude over 50 time points (about 1 cycle) at the location of L2. With the relative optical absorption spectrum of a calcaneus bone measured, spectral unmixing was then be performed to derive the relative contribution of each chemical component to the optical absorption spectrum. [29] A least-square method was used which tries to fit the PA-measured optical absorption spectrum of the bone via a linear combination of the optical spectra of all the relevant chemical components leading to the minimum sum of the squared residuals of the measurement points from the fitting line.

### 4.5. PA Spectral Analysis of Bone Microarchitecture

The PA spectral analysis methods developed recently by research groups including ours offer a new way for quantitative evaluation of the microstructures of the optically absorbing materials in biological tissues and have been explored for potential diagnosis and characterization of many pathological conditions [21, 37–39]. In frequency domain analysis of the power spectrum of the PA signal generated from a tissue and detected by a transducer, not only the microstructure of the tissue but also the ultrasound attenuation in the tissue affects the PA spectral analysis results. This is especially true in the bone where the ultrasound attenuation is high. In addition, because the ultrasound attenuation in the bone is highly dependent on the frequency, the compensation of ultrasound attenuation should be conducted for each frequency in the entire frequency range before PA spectral analysis can be applied.

We first converted the time domain PA signal to time-frequency domain by performing short-time Fourier transform to get the time-frequency spectrum of $F_{0}\left(t,f\right)$:

(11)F0t,f=STFTpt.

Then, the compensated time-frequency spectrum $F_{c}\left(t,f\right)$ was obtained by

(12)Fct,f=F0t,f∙10BUA∙f∙SOS∙t/20∙D−d1+d2.

**Figure 7:**
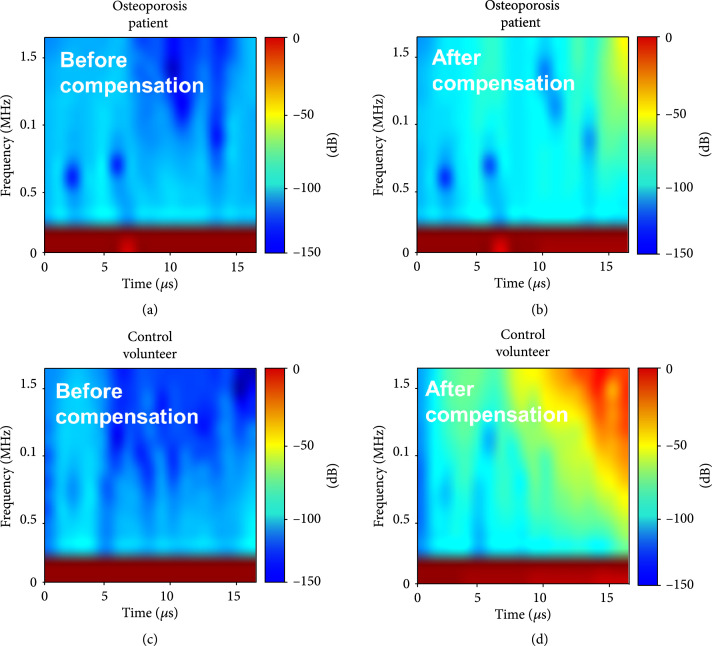
PA time-frequency spectra measured from the human calcaneus bones *in vivo*. (a, b) PA time-frequency spectra from the calcaneus bone of an osteoporosis patient before and after compensating the ultrasound attenuation. (c, d) PA time-frequency spectra from the calcaneus bone of a control volunteer before and after compensating the ultrasound attenuation.

The f in equations (11) and (12) was the frequency in the studied frequency range of 0-1.5 MHz. Typical normalized time-frequency spectra from the human calcaneus bones *in vivo* before and after the compensation of the ultrasound attenuation are shown in Figure 7, where the results from a control subject and an osteoporosis patient are compared.

With the compensated time-frequency spectrum of $F_{c}\left(t,f\right)$, the power spectrum density $\text{PSD}\left(f\right)$ was obtained by

(13)PSDf=∫Fct,f∙dt.

Then, the spectral analysis of the PSD was performed, following the methods described in detail in a previous publication. [33]

The PSD of the radio-frequency (RF) PA signal from each calcaneus bone was calculated and normalized at 0.1 MHz. Then, a linear fit of the PSD curve from 0.1 to 1 MHz was conducted, which led to a quantified spectral parameter *slope*. According to our previous studies, [21, 37] the spectral parameter *slope* is highly sensitive to tissue heterogeneity and the change in tissue microarchitecture. In addition, by integrating the normalized PSD curve (i.e., calculating the area under the normalized PSD curve) from 0.1 MHz to 1 MHz, a *weighted frequency* with a unit of dB·MHz was quantified as another PA spectrum parameter. The spectral analysis was conducted in the frequency range of 0.1-1 MHz as it was covered well by our transducer with a center frequency of 0.5 MHz. In addition, due to the strong acoustic attenuation in the bone, most of the PA signals received by the transducer are lower than 1 MHz. Setting the cutoff frequency at 0.1 MHz avoided the very strong low frequency noises. In this study, the PA spectral analysis was performed at two different laser wavelengths, including 800 nm and 930 nm. The 800 nm wavelength is the isosbestic point of the optical absorption spectra of oxy- and deoxy-hemoglobin [40] and thus is often used to present the content or the spatial distribution of whole blood. In contrast, the 930 nm wavelength corresponds to the strong optical absorptions of lipid. Intertrabecular pores are filled with bone marrow rich in both blood and lipid clusters, and hence, the measurements from PA spectral analysis at these two wavelengths can be used together to reflect the heterogeneous spatial distributions of lipid and blood in the trabecular bone as well as trabecular porosity.

### 4.6. Statistical Analysis

For each of the subject group, the 20 feet of 10 subjects were measured by DEXA and our PA system. Two feet from different patients (right foot of P004, left foot of P008) in the osteoporosis group were excluded for any statistical analysis of PA results due the low SNR (<4) of the PA signals. Two volunteers (H005, H008) from the control group were also excluded for any statistical analysis because the SNR of both feet were low (<4). For each subject, the quantified data of both feet were averaged before the statistical analysis. In total, N=10 osteoporosis patients and N=8 control volunteers were used for the statistical analysis of the PA results, while all the subjects (N=10) for each group were used for statistical analysis of the DEXA results.

The measurements used for statistical analysis were as follows: (1) the BMD results of the spine, total femur, femur neck, and femur upper neck, respectively, as measured by DEXA; (2) the relative optical absorption spectral curves calculated from multiwavelength PA measurements at 690-950 nm; (3) the average contents of different chemical components, including mineral (hydroxyapatite), deoxy-hemoglobin (Hb), oxy-hemoglobin (HbO_2_), and lipid, as well as blood oxygen saturation (SaO_2_), for both feet, which were unmixed from the curves in (2); (4) the PSD curves of the RF PA signals at the frequency range of 0.1-1 MHz; and (5) the quantified PA parameters including *slope* and *weighted frequency* for both feet by performing the PA spectral analysis on the curves in (4). The statistical analyses were performed to differentiate the control group and the osteoporosis group.

To evaluate whether the studies (1), (3), and (5) in the above can lead to statistical significant difference between the two subject groups, unpaired two-tailed independent sample t-tests (with Welch's correction in cases of unequal variances) were conducted by using the GraphPad Prism 7.0 software. To evaluate whether the studies (2) and (4) in the above can lead to statistical significant difference from the two subject groups, two-way ANOVA tests were conducted by using the GraphPad Prism 7.0 software.
